# Review on Sensor Array-Based Analytical Technologies for Quality Control of Food and Beverages

**DOI:** 10.3390/s23084017

**Published:** 2023-04-15

**Authors:** Abhishek Kumar, Mickael Castro, Jean-François Feller

**Affiliations:** Smart Plastics Group, Institut de Recherche Dupuy de Lôme, CNRS 6027, University of South Brittany (UBS), Rue de Saint Maude, 56100 Lorient, France; mickael.castro@univ-ubs.fr

**Keywords:** volatolomics, gas sensors, e-nose, food quality control, chemometrics, nanomaterials

## Abstract

Food quality control is an important area to address, as it directly impacts the health of the whole population. To evaluate the food authenticity and quality, the organoleptic feature of the food aroma is very important, such that the composition of volatile organic compounds (VOC) is unique in each aroma, providing a basis to predict the food quality. Different types of analytical approaches have been used to assess the VOC biomarkers and other parameters in the food. The conventional approaches are based on targeted analyses using chromatography and spectroscopies coupled with chemometrics, which are highly sensitive, selective, and accurate to predict food authenticity, ageing, and geographical origin. However, these methods require passive sampling, are expensive, time-consuming, and lack real-time measurements. Alternately, gas sensor-based devices, such as the electronic nose (e-nose), bring a potential solution for the existing limitations of conventional methods, offering a real-time and cheaper point-of-care analysis of food quality assessment. Currently, research advancement in this field involves mainly metal oxide semiconductor-based chemiresistive gas sensors, which are highly sensitive, partially selective, have a short response time, and utilize diverse pattern recognition methods for the classification and identification of biomarkers. Further research interests are emerging in the use of organic nanomaterials in e-noses, which are cheaper and operable at room temperature.

## 1. Introduction

**Figure 1:**
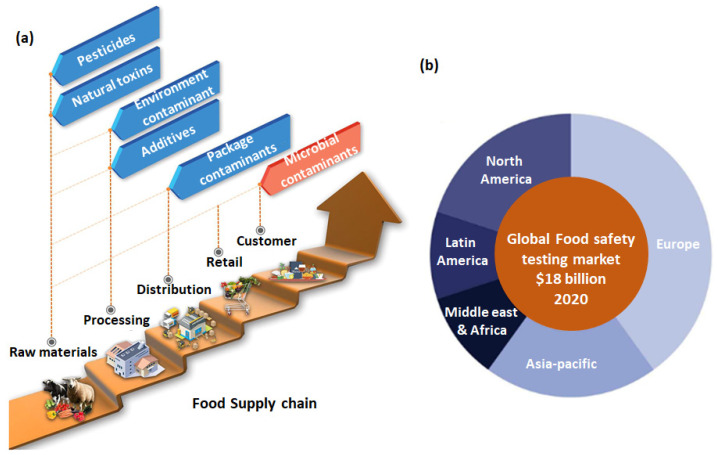
Scheme of the food supply chain and classification of potential contamination risks involved at different levels (**a**); adapted with permission from ref. [7]. schematic of global distribution in the food testing market (**b**).

The growing awareness about food safety is of paramount importance in the present globalized world, witnessing unprecedented trade of food supplies among countries, exponential growth in packaged food consumption, and rising cases of food adulteration. Indeed, food safety is a public health concern, which is also highlighted by the World Health Organization (WHO) in its recent report, which mentions nearly 600 million cases of illness and around 0.42 million deaths each year associated with contaminated food [1]. The developed countries, such as the USA, are also reporting more than 33 million foodborne illnesses and 9000 related deaths annually [2]. The potential risks of contamination in food products are present at every level of the food supply chain, as indicated in Figure 1a. Raw materials, such as livestock or agricultural products, can be contaminated by the excessive use of chemical pesticides and fertilizers, in addition to natural toxins present in the environment. Processing of the raw materials in a close industrial setting possesses the risk of overuse of unhygienic additives and contamination associated with a closed environment in the industries. Distribution and retailing of the food materials involve serious concerns arising from unscientific packaging, causing external contamination or rotting of particularly perishable foods, such as fish, meat, and green vegetables. Finally, on the customer’s plate, microbial contamination of the food is an eminent danger if served unhygienically. It is worth noting that in all levels of the food supply chain, “Economically motivated adulteration (EMA)” of food, which is also termed “Food fraud” by the Global Food Safety Initiative (GFSI), has drawn the attention of the authorities in recent years [3]. GFSI has defined EMA as an intentional practice of deliberate substitution, addition of ingredients in the food, tampering, misrepresentation, and providing misleading information on packaging labels for economic gain. Such fraudulent practices pose a serious health risk to consumers. In particular, fruit juices, mainly orange and apple juices, are among the most targeted packaged beverages for such adulteration and food fraud practices. Frequently, they are mixed with cheaper juices, sugar, and chemical additives and diluted with water [4]. These practices cause a loss of the food value that consumers are paying for and may cause severe allergic complications [5]. Thus, considering the multitudes of food frauds being practiced and the associated risks involved at every level of the food supply chain, the regular quality check of the food products is a prerequisite to assessing their authenticity and to reassuring the producers, food processing companies, and customers. Indeed, the quality check of food materials has been made mandatory by the government authorities at different levels of the food supply chain. According to European Commission-specific Community Directive 2012/12/EU and Council Directive 2001/112/EC, it is necessary for the manufacturer to report the composition of fruit juices, the concentration values, reserved fruit names, and any additional chemical additives on the label of the pack. In the same direction, the Association of the Industry of Juices and Nectars of the European Union (AIJN) has set a reference guideline to evaluate the quality, authenticity, and identification of fruit-based juices [6].

To implement the consumer health safety guidelines, food products require a regular quality assessment through reliable, cost-effective, and rapid analytical tools. Different analytical methods have been commercialized for food quality testing, including the conventional approaches based on chromatography and emerging methods based on point-of-care sensor technologies [8,9]. Owing to the stringent government regulations and consumers awareness about food quality, the food testing market has witnessed rapid growth in the last decade and had a market of $18 billion in 2020 [10]. Notably, the largest share is owned by Europe, and this market is expected to grow by over $40 billion in the next 10 years due to the burgeoning packaged food and online food delivery markets [11].

Herein, the objective of this manuscript is to perform a non-exhaustive review of the recent research progress reported in the literature about different analytical methods in food quality and authenticity assessment. First, we highlight the key parameters in food products, such as those based on its aroma and chemical properties, which are exploited in the different analytical methods. Our review methodology involves a comparison of the research in conventional analytical methods based on chromatography and spectroscopy and the emerging point-of-care technologies, such as gas sensors array based devices. For different analytical approaches, their key advantages and limitations are highlighted. Finally, a concise vision of the future research perspectives on improving the performances of analytical methods in food quality monitoring is presented.

The technical methodology of the review involved the use of leading search engines in the scientific domain, such as Google Scholar, SciFinder, Science Direct, and Scopus. In addition to these, the databases of AIJN, WHO, and the National Institute of Standards and Technology (NIST) were also consulted to find the government norms and recommendations in food quality control. The initial research was conducted using the keyword “analytical methods in food quality control”, in Scopus, which showed 373 results in the last 4 years, out of which 112 documents were open access. All the reported research involved scientific articles, reviews, book chapters, and conference papers. The research result was further refined by including “Sensors”, which increased the number of available documents to 94. A separate search was performed on Scopus using the keyword “electronic nose in food quality control”, yielding 160 results in the last four years, of which 61 were open access. Among these results, 110 were articles, 33 were reviews, 8 were conference papers, and 5 were book chapters.

## 2. Targeted Parameters in Food Quality Control

### 2.1. VOC-Based Parameters

**Figure 2:**
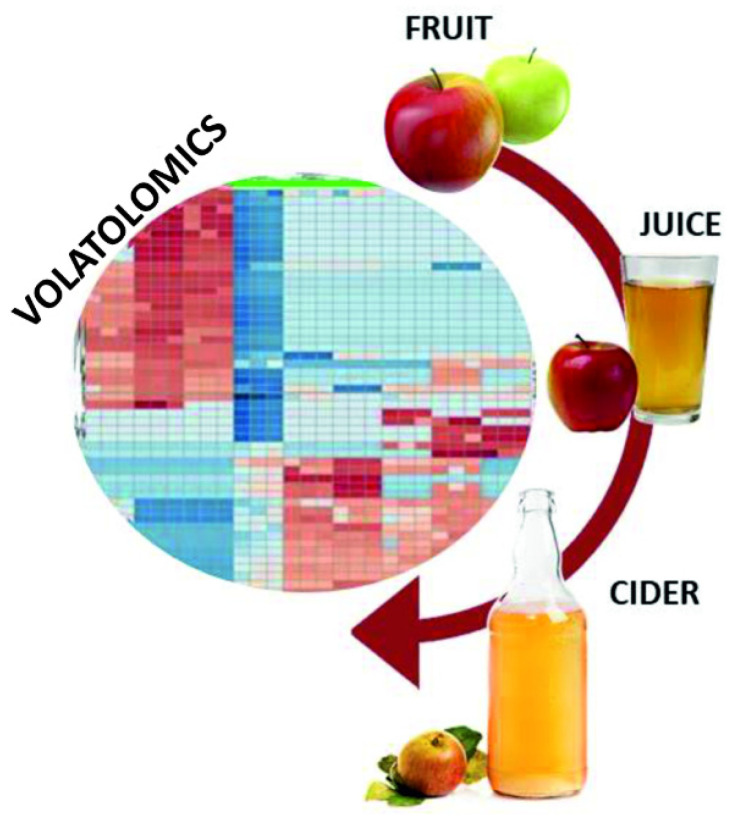
Scheme of VOC biomarkers assessment from the food products. (Adapted from ref. [14]).

Conventionally, the analytical methods used for the food quality assessment are based on targeted analysis, in which a set of predefined compounds or ions, typically called biomarkers, are searched in the detection process in the analyzed food samples. A sizable section of these biomarkers consists of volatile organic compounds (VOC), involving organic acids, esters, aliphatic alcohols, polyphenols, aldehydes, ketones, amino acids, and long-chain and cyclic alkanes. The presence, absence, and concentration of these VOC biomarkers constitute the food’s aroma. Thus, a change in the aroma of fruits, vegetables, meats, and fish corresponds to the alteration of the volatolomics profile of the associated food products. It may involve a change in the concentration of the already-present VOC in the fresh products or the addition of a new VOC because of the bacterial oxidation of the food during the spoilage [12,13]. Therefore, evaluating the VOC fingerprints of a fresh food and its rotten form can serve as the basis for real-time food quality monitoring during its storage period. Medina et al. [14] reported an extended list of VOC fingerprints present in different varieties of apples and their derived juices and ciders in fresh and fermented forms (Figure 2). The study revealed that the natural apple aroma mainly consisted of VOCs of the ester, aldehyde, ketone, organic acid, and alcohol families.

From these groups, the predominant compounds in fresh apples aroma were 2-methyl butyl acetate, butyl acetate, and 2-hexenal. However, during the bacterial fermentation, additional VOCs, such as ethyl hexanoate, ethyl octanote, 3-methyl-1-butanol, 2-phenylethanol, and ethyl decanoate, were formed. Buron et al. [12] listed different polyphenols produced in apple cider as a consequence of bacterial and yeast fermentation. Volatolomics profiles of different Muscadine grape varieties were assessed by Deng et al. [15] through solid–phase microextraction coupled gas chromatography, identifying 44 unique VOCs from the families of ester, ketone, terpene, organic acid, aldehyde, alcohol, fatty acid, and furan. Different VOC biomarkers in fresh and sweet orange and mandarin juices were identified by Zhou et al. [16], which contained compounds from the group of monoterpenes, aldehydes, ketones, esters, sesquiterpenes, and alcohols. The fingerprinting revealed 20 unique VOCs present in sweet orange juice and 9 unique VOCs present in mandarin juice, thus setting a basis for their identification and recognizing any possible adulteration by mixing these two juices. Villatoro et al. [17] monitored the change in aroma and the associated VOC biomarker blend of the “Pink Lady” apple variety during different stages of its ripening. It was found that VOC content was significantly low in unripe fruit, while the content of hexyl acetate, hexyl hexanoate, hexyl 2-methylbutanoate, hexyl butanoate, 2-methylbutyl acetate, and butyl acetate significantly increased as ripening advanced. Thus, the strategy served as a highly useful tool for classifying different types of apples and segregating the rotting fruit. 

The spoilage of orange juice over time under different storage conditions was monitored by de Oliveira et al. [18] by tracking the volatolomics profile through NMR spectroscopy combined with chemometric analysis. It was found that the concentration of certain VOCs, such as formic, acetic, fumaric, succinic, and lactic acid, significantly increased after 24 h of storage, in addition to the detection of ethanol, which was absent in the fresh orange juice. Moreover, the production of these biomarkers was more notable when storing juice at 24 °C, compared to storage under a fridge. Nuncio-Jáuregui et al. [19] detected adulteration of pomegranate juice, which is relatively expensive, by the mixing of comparatively cheaper grape and peach juices, through monitoring the change in the VOC profiles. The adulteration with 10% (*v*/*v*) of grape juice resulted in a significant rise in 1-hexanol and linalool, in addition to acetic acid and isoamyl butyrate. On the other hand, adulteration with 10% (*v*/*v*) of peach juice caused a significant increase in isoamyl butyrate in addition to butyl acetate, isobutyl butyrate, and benzyl acetate. Orange juice is the most consumed fruit juice globally, so detection of its possible adulteration through VOC biomarkers has drawn a lot of attention. Schmutzer et al. [20] performed a comprehensive analysis of VOC content in 23 different types of commercial orange juice and proposed a flavor index to detect possible adulteration by dilution with water. The proposed flavor index corresponds to the ratio of total sesquiterpenes to total terpenes (without limonene) in the juice sample. A benchmark value of 40% is set for standard orange juice, while any upward or downward shifts from this value indicate concentrated or diluted juice, respectively. Moreover, a higher content of total ketones and total alcohols was detected in the commercially processed orange juice compared to freshly prepared homemade orange juice. The study by Bocharova et al. [21] reported the detection of synthetic food flavor additives in commercial orange juice by identifying high concentrations of benzoic acid and D-limonene, which were absent in the freshly squeezed juice and the one prepared without the additives. Rothwell et al. [22] reviewed the recently reported literature on VOC biomarker analysis in coffee, tea, and other non-fruit sweetened beverages. It was reported that coffee contained mainly VOC compounds derived from phenolic acid, alkaloids, and terpenes, with high specificity for trigonelline and cyclo(isoleucylprolyl), while black tea and green tea reveal specific biomarkers as 4-O-methylgallic acid and epigallocatechin, respectively. From these examples, it is evident that identifying VOC biomarker emissions in food products at different stages is useful in predicting their quality. Some of the commonly observed VOC fingerprints assessed by different analytical tools in fruits, vegetables, and packaged fruit beverages are listed in Table 1. It has to be noted that the mentioned compounds are not an exhaustive list of all the VOCs present in the respective food products but correspond to the major VOC species detected among hundreds of other VOC biomarkers. Moreover, VOC profiles change depending on the geographical origin of fruits and vegetables, their storage conditions and processing during industrial manufacturing, such as thermal heating or the addition of preservatives.

### 2.2. Non-VOC-Based Parameters

Apart from the organoleptic feature of the food aroma, other parameters, such as pH, Brix value, formol number, ascorbic acid content, individual amounts of particular ions such as K^+^, Na^+^, Mg^2+^, Ca^2+^, total O_2_ and CO_2_ content, humidity, and specific chemicals related to food types are also taken into account to predict the possible adulteration or quality degradation in food products. The brix value/index of a fruit juice indicates the total dissolved sugar in grams per 100 mL of its volume, which is also proportional to its specific gravity [45]. The adulteration of a commercial fruit juice through the addition of external sugar or dilution with water can be detected by an increase or decrease in its brix value, respectively, from the standard value evaluated for a 100% pure juice. The formol index of any fruit juice represents the total amino acid content, which is estimated against titration with NaOH solution in formaldehyde [46,47]. The increase in the formol index of a fruit juice indicates a possible adulteration with cheaper artificial additives. Lorente et al. [48] performed a comprehensive analysis of 184 fruit samples, determining the brix and formol indexes, ascorbic acid content, metal ions, and organic acids, which were found to be below the maximum permissible limit according to AIJN guidelines. The individual content of a specific metal ion and the ratio of two metal ions have been used as markers to detect potential adulteration in different fruit juices. For instance, low K^+^ content in commercial orange juice often indicates overdilution with water, as oranges are characterized by their high K^+^ content [20], while high Na^+^ content indicates the presence of artificial preservatives or synthetic flavor additives. Moreover, high Ca^2+^ concentration in the orange juice is a signature of adulteration with orange pulp because pulp wash contains more Ca^2+^ than the juice. Similarly, a K/Mg ratio of less than 50% in commercial orange juice is a marker of the presence of artificial sweetener [49]. In the case of commercial pineapple juice, which often encounters adulteration with cheaper grape or peach juice, K^+^ content lower than 2000 mgL^−1^ indicates the occurrence of such adulteration [19]. 

## 3. Different Analytical Methods in Food Quality Assessment

### 3.1. Conventional Methods Based on Passive Sampling

The aforementioned parameters/markers to evaluate the food’s quality can be detected through multitudes of analytical techniques. The conventional approach involves the passive sampling of the targeted VOC biomarkers or other detectable parameters, which are subsequently analyzed in laboratories. The most commonly used analytical approach is based on chromatography, such as high-performance liquid chromatography (HPLC), gas chromatography (GC), and thin layer chromatography, which are used in tandem with a mass or UV-Vis spectrophotometer as detectors. Other than chromatography-based methods, spectroscopy, electrophoresis, DNA-based techniques, and elemental analyses are also employed. Notably, all the analytical methods use advanced chemometrics tools and comparison with a standard database to improve the signal discrimination of the targeted markers.

#### 3.1.1. Chromatography-Based Approaches

**Figure 3:**
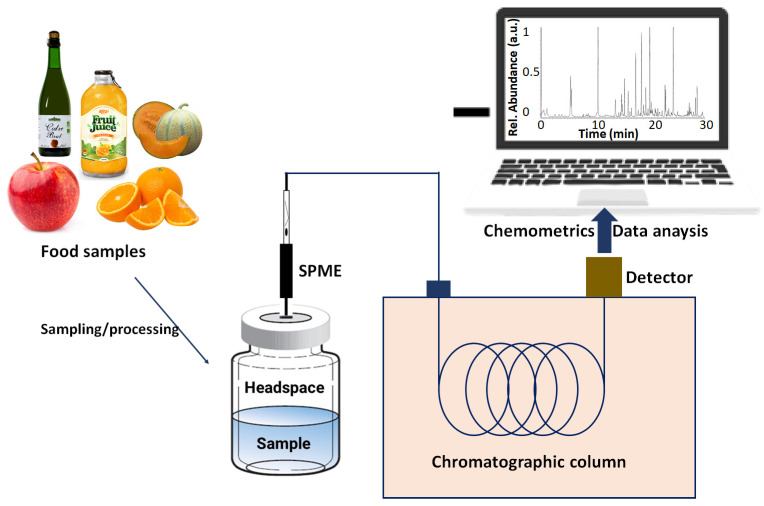
Schematic illustration of food sample analysis by gas chromatography coupled with a mass spectrometer detector and chemometrics data treatment.

Chromatography-based approaches employ the Headspace-Solid Phase Microextraction (HE-SPME) sampling method [50] to extract the VOC blend, which is further analyzed to identify the different groups of VOC biomarkers and their concentrations. A typical analysis of a food sample by gas chromatography is depicted in Figure 3. Herein, a raw food sample is first processed (grinding/filtering), followed by incubation in a suitable container. The volatile compounds tend to accumulate above the liquid surface and establish a dynamic equilibrium, owing to low saturation vapor pressure, a mixture of which is typically referred to as “headspace” [51]. The accumulation of headspace vapor can be further fastened through thermal heating or bubbling a flow of dry air inside the liquid. The volatile vapors in the headspace are then collected through SPME, followed by their transfer through the pump into the chromatography column, where they are separated owing to differences in the interaction with the column material and the saturation vapor pressure/boiling point of the constituent VOCs in the mixture. The ejected vapors are then analyzed as a function of time with a suitable detector, such as a mass, UV-Vis, or infrared spectrometer, generating peaks characteristic of each VOC present in the headspace. The peaks and their area are finally analyzed and compared with the standard database using advanced statistical and mathematical tools to identify each VOC present and its concentration in the analyzed sample.

**Figure 4:**
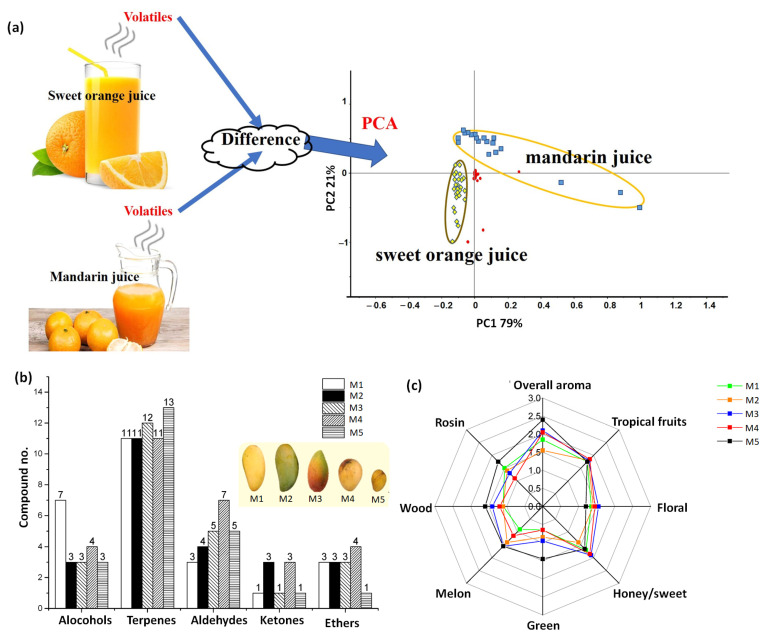
The detection of adulteration in sweet mango juice with mandarin juice by PCA. PCA score plot, depicting sweet orange juice, mandarin juice, and blend samples marked with green, blue, and red dots, respectively (**a**). Adapted from ref. [16]. Comparison of the bar plots of the different VOCs present in five mango samples (**b**). Adapted from ref. [58]. The spider plot showing the assignment of different aromas to each mango variety (**c**). Adapted from ref. [58].

Zhou et al. [16] used the GC/MC method coupled with multivariate data treatment by Principle Component Analysis (PCA) (Figure 4) to detect the adulteration of sweet orange juice by mandarin juice. The VOC profiling g revealed more than 32 species, of which many were common in both juice samples. The PCA data treatment could discriminate between the signals associated with sweet orange juice and mandarin juice, as indicated in Figure 4, indicating that the principle components of each constituent and the blend are resolved. However, below 10% of adulteration with mandarin juice could not be accurately detected. Abad-Garcia et al. [52] reported a reversed-phase HPLC method to evaluate the quality of 18 different types of fruit juices from 9 different fruits, revealing 55 different types of polyphenols of the classes hydroquinones, flavonols, flavanones, coumarins, flavones, and hydroxycinnamic acids. The low detection limit of these polyphenols, assessed in the range of 0.005 to 0.03 μg.cm^−3^, combined with high selectivity make the measurement platform highly efficient in monitoring the quality of packaged fruit juices over time. Wang et al. reviewed recent progress in different types of chromatography-based methods for detecting VOC biomarkers associated with Carbendazim fungal infection in different food samples (fruits, vegetables, meats, fish, baby foods, nuts, juices, honey, and flours) [53]. Elsewhere, the use of thin layer chromatography was reported for the quality assessment of alcoholic and non-alcoholic beverages derived from fruits [54,55]. The methods generated a chemical profile of different VOC biomarkers and other parameters (polyphenols, carboxylic acids, dyes, amines, and ascorbic acid) to evaluate the authenticity of the beverages. The presence of an artificial food additive, morpholine, which is used to impart extra shine to fruits and vegetables, was detected by the gas chromatography-tandem mass spectroscopy method by Han et al. [56]. The method was highly sensitive, with a limit of quantification of 10 µg.kg^−1^, selective, and accurate. The packaged fruit juices on the market are made either by squeezing the fruit or by diluting the solid fruit concentrate, making their authentication a big challenge. An innovative methodology based on ultra-fast gas chromatography analysis was reported by Rozanska et al. [57] to identify VOC biomarkers present in pure orange and apple juice made “not from concentrate”. The authors used multiple chemometric tools, such as Classification Tree, Neural Network, Hierarchical Cluster Analysis, Naïve Bayes, and Random Forest classifiers, to discriminate signals associated with each VOC biomarker. The method was highly sensitive, as it could identify the presence of impurities/adulteration up to 1%.

Liu et al. employed SPME-coupled gas chromatography and a mass spectrometer detector to identify the aroma of mangoes from five different geographical origins [58]. Different mango varieties revealed mainly the presence of alcohols, terpenes, esters, aldehydes, and ketones group of VOCs (Figure 4b), in which the origin of the unique aroma in each variety was attributed to 1-hexanol, γ-terpinene, hexanal, trans-2-heptenal, and p-cymene. Based on the chemometric analysis using SPSS statistics on the intensities of different VOC in the mangoes, their aroma was classified into seven different types through a spider plot (Figure 4c), differentiating each mango variety by its unique aroma. The work by Rinaldi et al. proposed a new chemometric approach to differentiate the authenticity of freshly squeezed orange juices derived from oranges belonging to four different geographical origins [59]. The SPME–GC-MS analysis revealed the presence of VOCs belonging to mainly monoterpene and sesquiterpene hydrocarbons, alcohols, aldehydes, and esters groups in oranges derived from different cultivars. The discrimination of signals corresponding to each orange variety was achieved with high accuracy by the combination of Linear Discriminant Analysis (LDA) pattern recognition, the genetic algorithm, and the Kohonen map.

#### 3.1.2. Spectroscopy-Based Approaches

**Figure 5:**
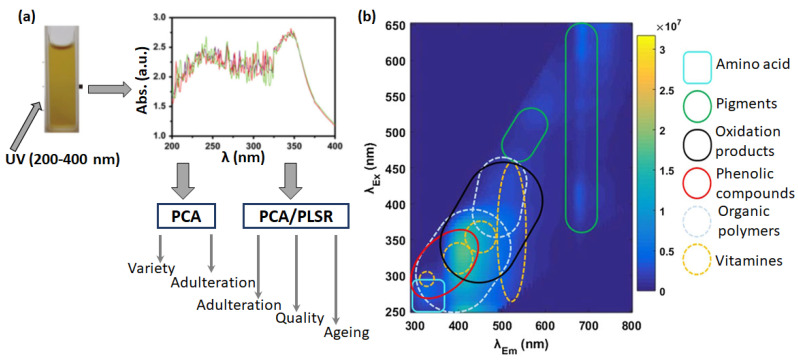
Schematic illustration of apple juice analysis by UV-Visible absorption spectroscopy and analysis of its data by chemometrics to predict variety, adulteration, quality, and ageing. The spectra of the juices derived from four different apple varieties are compared in different colors. (**a**). Adapted from ref. [60]. Mapping of fluorophores present in different chemical compounds in a fruit sample, performed by front-face fluorescence spectroscopy with excitation wavelengths of 250–650 nm and emission wavelengths of 290–800 nm. The region with a dark blue color indicates absence, while the region with a yellow color marks the presence of fluorophores, respectively (**b**). Adapted from ref. [66].

Spectroscopy-based analytical methods, such as UV-Visible absorption, fluorescence, and vibrational spectroscopies, are another widely used conventional approach to evaluating food authenticity. They are comparatively less expensive, simpler to use, and faster than the chromatography-based analytical methods, which do not require complex sample pretreatment before the analysis. The recent study by Chang et al. [60] proposed a simple approach based on UV-Vis spectroscopy coupled with chemometrics (Figure 5a) to detect the geographical origin, possible adulteration, quality, and ageing of apple juice. The combination of absorbance data and PCA could accurately identify the apple varieties and detect adulteration up to 0.8%. The nutritional quality of the juice in terms of soluble solids, total flavonoids, ascorbic acid, and total sugar as well as the ageing of stored juice were accurately predicted by combining UV data with partial least-squares regression (PLSR). Additionally, analysis of the UV data with principal component regression (PCR) revealed accurately the pH and total antioxidant activity of the juice sample. Boggia et al. [61] proposed a rapid approach based on UV-Visible spectroscopy and PCA multivariate exploratory data analysis to screen the commonly used artificial additives in the adulteration of pomegranate juice, which are responsible for decreasing the radical scavenging function of the juice. Recently, Farag et al. [62] reviewed the application of UV-Visible spectroscopy in food quality control and emphasized the advantages of chemometrics analysis on the measured absorbance and wavelength of the chromophores to generate a fingerprint of the different biomarkers and other parameters.

Fluorescence spectroscopy is a highly sensitive and selective technique capable of providing the composition, nutritional values, and possible adulteration of food by identifying the fluorescence emission and quenching of the fluorophore’s functionalities in the food. Karoui et al. [63] reviewed applications of fluorescence spectroscopy in combination with multivariate data analysis to predict the quality and authenticity of a wide range of food products (vegetables, fruits, meats, eggs, and cereals). Recent studies by Ammari et al. [64] and Wlodarska et al. [65] reported the use of 3D fluorescence and synchronous fluorescence spectroscopies in combination with chemometric tools (Independent Components Analysis, ICA; and Partial Least Squares Discriminant Analysis, PLS-DA) to detect adulteration and confirm the authenticity of orange and apple juices. Latchoumane et al. [66] reported a highly innovative approach to chemically map the whole fruit by 3D front-face fluorescence spectroscopy to detect unique fluorophores corresponding to different constituents in the fruit. The objective of the study was to predict any disorder caused by plant stress and infections and the extent of ripening of the fruit. For this purpose, the fluorescence emission wavelengths were analyzed by 3-way feature extraction using the N-CovSel chemometric method to generate a chemical/nutrition profile, such as amino acids, pigments, phenolic compounds, and vitamins present in the interior of the fruit (Figure 5b). Elsewhere, the application of fluorescence spectroscopy in combination with the Partial Least Squares (PLS) statistical method was demonstrated to classify twenty-seven apple juices derived from apple fruits of different geographical origins [67]. 

Infrared spectroscopy is another widely used optical spectroscopy method in the quality evaluation of food. It provides structural information about the molecules present inside the food sample based on the vibrational stretching of different chemical functional groups, thus generating a chemical fingerprint of the analyzed food. In recent years, a host of researchers have applied Fourier transform infrared spectroscopy (FTIR) characterizations in conjunction with advanced chemometrics analysis to classify different fruit beverages and evaluate their qualities [68,69,70,71,72,73,74]. Vardin et al. [69] reported FTIR analysis in ATR mode in combination with PCA data analysis to authenticate pomegranate juice and detect possible adulteration with grape juice. The key difference between pomegranate and grape juice is noted in the region 1780–1685 cm^−1^, which is attributed to C=O stretching. The very recent studies of Junges et al. [75] are notable, reporting a green analytical methodology that involved solvent-free FTIR analysis of conventional and organic grape juices. The two types of grapes contain different sets of polyphenols and organic acids, which are identified in the FTIR measurements, and a precise classification was achieved by the multivariate statistical methods LDA and PLS-DA. Wlodarska et al. [76] reported a comparative analysis by UV-Vis, fluorescence, and FTIR spectroscopies in combination with PLS data analysis to predict the quality of apple juice by rapidly screening parameters such as pH, titrated acidity, and soluble solid content. In addition to conventional optical spectroscopies, the application of Nuclear Magnetic Resonance (NMR) spectroscopy in conjunction with PCA chemometric analysis was also demonstrated to monitor the degradation of fresh orange juice and assess the geographical origin of oranges [18].

### 3.2. Sensor-Based Methods for Real Time Analysis

Despite the high sensitivity, unique selectivity, and accuracy of measurement demonstrated by the aforementioned conventional analytical methods, there are challenges, some of which are intrinsic to the methods of measurement. The key disadvantage of these methods is the requirement for passive sampling of the targeted biomarkers and other parameters, which bears the risk of contamination/transformation/leak of the sampled headspace while being transported to the laboratory. In addition to this, these methods are expensive, time-consuming, and require skilled analysts to perform the measurements. To overcome these limitations, sensor-based analytical platforms are highly promising, owing to their miniaturized form, lower cost, ease of operation by unskilled users, possibility to tune the metrological parameters by the change of the sensing materials and transducers, and ability to mount multiple sensors into an array to perform multiplex measurements. The strategy of using an array of sensors coupled with chemometrics has been extensively researched in recent years in the area of food quality assessment. These sensors are commonly referred to as electronic tongue (e-tongue) or electronic nose (e-nose) [77,78], depending on whether they measure taste (commonly in liquid) or odors (usually in gas medium), respectively.

**Figure 6:**
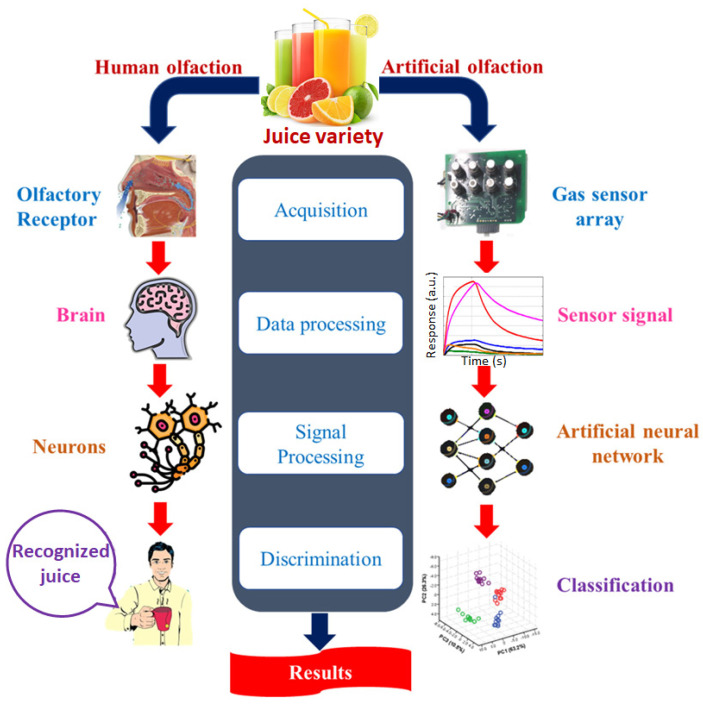
Schematic representation of an e-nose device and comparison of biological olfaction by the human nose and artificial olfaction by the e-nose. Adapted from ref. [89].

Herein, the focus of this section will be to report the recent progress made in e-nose advancements in identifying biomarkers in food to predict their quality. e-nose is a portable electronic device that mimics the sense of smell and thus impersonates the human nose in identifying the aroma of a food product, which is usually a combination of different VOCs. The e-nose can be more versatile than the human nose in terms of sensitivity and selectivity, as there are many VOCs for which the human nose lacks receptor neurons. The basic configuration of an e-nose, as depicted in Figure 6, consists of an array of gas sensors responsible for detecting different VOCs in the targeted aroma, which are connected to a suitable signal acquisition setup. The experimental data/signal is further processed through a pattern recognition algorithm, such as multivariate data analysis (PCA, LDA, and PLS) or network analysis (artificial neural network, radial basis function), to achieve discrimination of a VOC or group of VOCs in the aroma and identify the targeted VOC in the sample. The artificial olfaction operation of the e-nose is analogous to the biological olfaction of the human nose, which is also compared in Figure 6. The application of e-nose in food quality inspection has been demonstrated by many researchers, incorporating different types of gas sensors and pattern recognition chemometrics modules [9,79,80,81,82,83]. A sizable chunk of these devices, based mainly on metal oxides-based semiconductor gas sensors, has also been transferred from the laboratory benches to the market shelves. For instance, Alpha MOS in France provides the commercial e-nose of models FOX 3000 and FOX 4000, incorporating 6 metal oxide semiconductor (MOS)-based sensors in the former and 18 MOS in the latter. These devices have been successfully implemented in monitoring the degradation of peach juice over time during its storage [84] and defining the aroma of coffee beans during the roasting and drying processes [85]. Airsense Analytics in Germany offers miniaturized, thus portable, PEN 2 and PEN 3 e-nose systems [86], utilizing arrays of 18 and 10 MOS-based sensors, respectively, whose operational efficiency has been demonstrated in recent studies to inspect the quality of litchi fruits stored in different environments [87]. The gas sensors component in the majority of e-nose devices developed for the food quality assessment are based on resistive/conductometric transducers [88], utilizing metal oxides and conducting polymer-based nanomaterials, which are discussed in detail in the subsections hereinafter.

#### 3.2.1. Metal Oxide Semiconductor-Based E-nose

**Figure 7:**
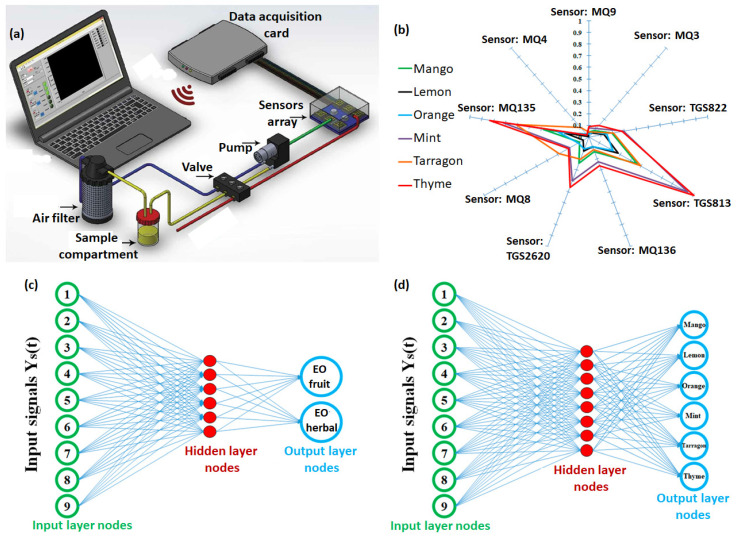
Schematic representation of the MAU-9 e-nose measurement set up, consisting of 9 MOS-based chemiresitive gas sensors, fluidic connections, a data acquisition card, and a computer equipped with pattern recognition software (**a**). Radar plot, depicting the response of each sensor in the array towards the VOC mixture extracted from different essential oils (**b**). Discrimination of observed sensor response by an artificial neural network into two groups, essential oils derived from fruits and herbs (**c**); and into six groups, derived from each source (**d**). Adapted from ref. [91].

Undoubtedly, MOS materials, such as SnO_2_, TiO_2_, ZnO, and In_2_O_3_, have been most widely used in gas sensor technologies, and many of them have been commercialized. These sensors work on the principle of resistance/conductance variation of the metal oxide sensing material upon chemical doping by the exposed gas molecules [90]. It causes a change in the mobile charge carriers’ concentrations (electrons or holes) in the conduction or valence band of the semiconducting material, depending on its n-type or p-type as well as the oxidizing or reducing nature of the gas. The application of MOS-based e-nose in detecting fruit juice adulteration and essential oil fraud was recently reported by Rasekh et al. [91,92]. They utilized a commercial MAU-9 e-Nose module, consisting of an array of nine MOS gas sensors, a data acquisition card, and statistical and network analysis software that was integrated into a gas sensing platform (Figure 7a). The test aroma, comprising a VOC mixture, was collected from the dynamic headspace created by bubbling a fixed air flow in the sample compartment, which was further passed through the array of MOS sensors. The response of nine MOS sensors in the array towards the aroma of six different essential oils, derived from fruits (mango, lemon, and oranges) and herbs (mint, tarragon, and thyme), was converted into a radar plot as shown in Figure 7b. The radar plot reveals different sensitivity of each MOS sensor towards the six tested aromas, such that sensors had a smaller response towards fruit-based aromas than herb-based aromas. Moreover, sensors referenced as MQ135 and TG813 displayed a much higher response than others, whereas sensors MQ3, MQ4, and MQ9 showed a very low response. Such classification of the e-nose response during the training phase (known as aroma) allows to choose the sensors, exhibiting higher response and better selectivity, which facilitates the simplification of the statistical or network data analysis and improves the discrimination of the signals. Observed signals from nine MOS gas sensors to six aromas were analyzed with an artificial neural network (Figure 7c,d). The analysis could classify the aroma associated with essential oil into two groups, derived from either fruits or herbs, with very high accuracy. However, the accuracy of the analysis is lowered when the output classification of aromas is made into six groups, discriminating the aroma of each of the six essential oils. 

**Figure 8:**
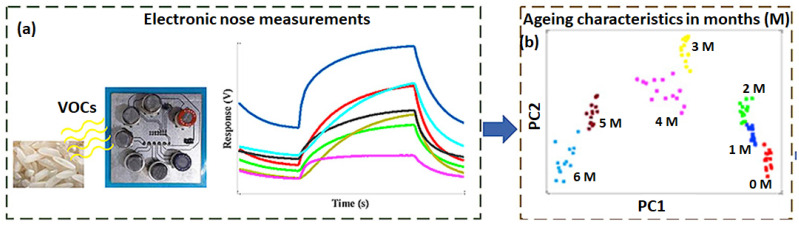
Schematic of rice aroma sensing by MOS gas sensors array and the observed response curves of the sensors in the array (**a**). A PCA score plot of the different aromatic samples collected for a storage period between 0 months and 6 months is shown (**b**). Month is represented as M in the graph. Adapted from ref. [100].

Berna et al. reported a comprehensive review of MOS gas sensors-based e-nose and their applications in quality monitoring of different types of food, such as fruit juices, edible oils, dairy products, eggs, fish, and meat [93]. Very recently, Teixeira et al. demonstrated the application of an e-nose, consisting of an array of nine commercial MOS sensors, in classifying extra virgin olive oil [94]. The classification was based on the intensity of their fruitiness aroma, originating from VOC biomarkers (acetic acid, hexenyl, 3-hexenol, 1-hexenol, hexanal, and nonanal), which were discriminated by PCA and LDA statistical analysis. In addition to this, MOS gas sensors-based e-nose have been recently investigated for differentiating cumin seeds based on their geographical origin [95] and the quality evaluation of peanuts [96], rapeseed [97], and onions [98]. Another notable application of MOS gas sensor-based e-nose has been monitoring the ageing of rice during its storage period [99,100] by evaluating the change in VOC pattern in the aroma. The studies performed on seven different types of rice varieties (e.g., White, Brown, Black, Basmati, Jasmine, Indica, and Japonica) revealed a rapid change in the concentration profiles of 2-acetyl-pyrroline, aldehydes, heterocycles, and alcohol VOC biomarkers. The e-nose employed for the studies consisted of seven commercial MOS gas sensors, a static headspace sampling unit, and a pattern recognition module (multivariate statistical and network analyzer). The sensor array was exposed to aroma collected from rice samples stored for different periods of time between 0 and 6 months. The dynamic response of seven sensors in the array towards the aroma collected from a rice sample stored for six months is shown in Figure 8a. The magnitude of the response and adsorption/desorption profiles of the sensors were found to be different. Analysis of the sensors data by PCA could accurately discriminate the aroma corresponding to each storing period of the rice, which is evident in the score plot in Figure 8b, depicting well-separated zones of the principal components.

#### 3.2.2. Organic Nanomaterial-Based E-nose

**Figure 9:**
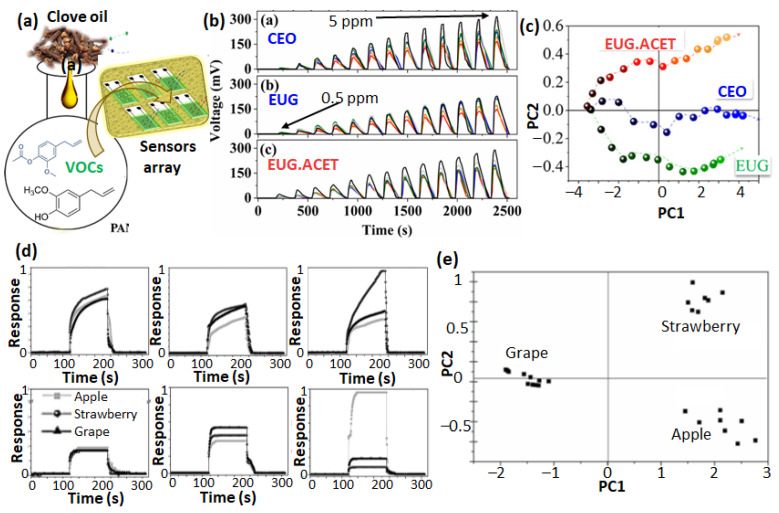
Representation of VOC biomarkers for clove oil detection by a CPC sensor-based array (**a**). The responses of the six sensors (indicated in different colors) towards three different VOC aromas in the concentration range from 0.5 to 5 ppm (**b**). PCA analysis of the e-nose signal towards three different VOCs (**c**). Adapted from ref. [118]. Individual response of the 6 poly(aniline)-based CPC sensors towards the aroma of apple, strawberry, and grape (**d**) and the corresponding score plot obtained after PCA analysis on the sensor response (**e**). Adapted from ref. [119].

Although MOS-based sensor arrays are highly sensitive to the VOC biomarkers commonly found in food quality evaluation, their operational requirement of higher temperatures makes them expensive to use. Moreover, MOS have limited selectivity for the VOCs belonging to the same functional family. These limitations have been addressed to some extent by organic nanomaterials-based gas sensors arrayed in the e-nose, which are relatively cheaper to synthesize and compatible with different deposition methods to obtain a homogeneous coating on sensor electrodes (solution casting, electrodeposition, and physical vapor deposition) [101,102]. Moreover, it is possible to tailor the material structure and properties to optimize the gas sensing performances [103,104,105]. Three types of organic nanomaterials have found significant research interest in chemiresistive/conductometric gas sensors: conducting polymer nanocomposite (CPC) [106,107,108,109], organic molecular materials (porphyrin, phthalocyanine, and corroles) [110,111,112,113], and nanocarbon materials (graphene, CNT, and fullerene) [114,115,116,117]. The application of CPC gas sensor-based e-nose in food quality analysis has been recently demonstrated by Graboski et al. [118,119]. In one of these studies, the e-nose device was used in evaluating the purity of clove essential oil (CEO) by identifying the key biomarkers eugenol (EUG) and eugenyl acetate (EUG.ACET) (Figure 9**a**) [118]. The sensing array consisted of six different CPC sensors, of which three were based on multiwall carbon nanotube/poly(aniline), while the other three had graphene oxide/poly(aniline)-based nanocomposite (each composite doped with three different dopants). The responses of the different sensors measured in the concentration range from 0.5 to 5 ppm were highly reversible, rapid, and specific for each sensor. For each VOC tested during the training stage of e-nose, a limit of detection (LOD) below 1 ppb and a response time of ca. 13 s were assessed. The analysis of the sensors responses with PCA could accurately classify the features associated with EUG, EUG.ACET, and CEO, which is evident in the score plot in Figure 9c. 

**Figure 10:**
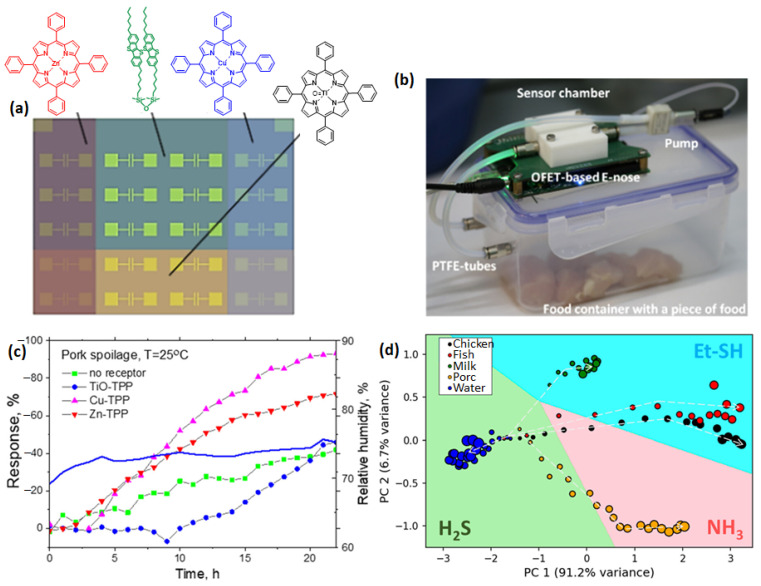
Schematic representation of metal (Zn, Cu, and TiO) porphyrin-based FET sensor arrays (**a**) and a real photograph of an e-nose measurement setup for meat samples (**b**). Comparison of the relative response evolution of different FET sensors towards the aroma collected from a pork sample at different storage times. The variation of relative humidity is indicated in solid blue line (**c**). PCA score plot of the aroma classification of different food products into different biomarkers (**d**). Adapted from ref. [121].

The same group of authors used poly(aniline) sensing layer-based sensors in the e-nose to characterize the aromas of apple, strawberry and grape [119]. Here, the polyaniline film was coated by two different methods: in situ adsorption polymerization and layer-by-layer deposition on gold interdigitated electrodes and printed graphite interdigitated electrodes, which were also doped with various dopants, forming six different sensors in the array. The response curve of each gas sensor towards the aroma of the three different fruits is depicted in Figure 9b, revealing a strong dependence on the poly(aniline) film type and the type of electrode on the response magnitude and adsorption/desorption kinetics. The sensors based on a printed graphite electrode were more sensitive, while those based on a gold interdigitated electrode showed a lower LOD. The analysis of the sensor’s response with PCA could classify the aroma of the three fruits, as evidenced in the score plot in Figure 9e. An e-nose containing CPC-based sensor arrays has also been employed to monitor the spoilage of dairy and meat products. Tung et al. reported CPC sensors array-based e-nose for the classification of VOC biomarkers commonly observed during meat spoilage, such as acetone, toluene, methyl acetate, ethanol, and dimethylsulphide [120]. The CPC material was prepared by dispersing reduced graphene oxide nanofiller in polymer ionic liquid and PEDOT polymer matrix. The sensors displayed a very short response time (3 s) and high sensitivity to the targeted VOCs, which were accurately discriminated by PCA data analysis. The application of metal porphyrins-based organic field effect transistors (OFET) in e-nose was demonstrated in identifying the biomarkers released during the spoilage of different meat and dairy products [121]. The four different arrays of FET sensors were realized by depositing a monolayer of a siloxane derivative of 1-benzothieno-benzothiophene by Langmuir-Blodgett (LB) deposition, which was the reference sample (Figure 10a). Three of the FET sensor arrays were coated with additional monolayer of zinc, copper, and titanium oxide tetraphenyl porphyrin (TPP) to impart selectivity in the sensor response. The e-nose containing the four different FET sensor arrays was tested for aromas produced at different storage times of meat and dairy samples (Figure 10b). The evolution of the responses of the four FET sensors towards the aroma of a pork meat sample at different storage times is shown in Figure 10c. Notably, the response of all the sensors increases for the aromas collected with increasing storage time, although each sensor displays a different magnitude of responses and variation patterns. The data associated with observed sensor responses were analyzed with PCA and vector comparison methods to predict the dominant biomarkers (Figure 10d).

## 4. Conclusions and Perspectives

In summary, the advancement in food quality assessment by different analytical techniques at the various levels of the food supply chain has been highlighted in the light of consumer health safety and the implementation of government quality control regulations. Such monitoring is important to authenticate the food qualities, which can be compromised either by intentional adulteration or degradation because of ageing. Moreover, it also helps to determine the geographical and varietal origin of the food, which is important to protect the claimed food quality by the producers. The majority of analytical methods used in the food quality evaluation are based on targeted analyses, identifying certain VOC biomarkers, pH values, Brix and Formol indexes, and the presence of certain metal ions ratios (K^+^, Na^+^, and Ca^2+^) to predict the quality of the food. Among them, VOC biomarker-based analyses, commonly termed “volatolomics” have gained a lot of research interest. The common VOC biomarkers found in the plant-based food products, such as fruits, vegetables, and their juices, involve the family of esters, aliphatic and aromatic alcohols, long-chain hydrocarbons, organic acids, terpenes, aldehydes, and ketones. Each aroma of a food has a unique combination and concentration profile of VOC biomarkers that predict the authenticity and age of the analyzed food sample. The conventional analytical methods are based on passive sampling of biomarkers from the food samples through static or dynamic headspace collection, which are subsequently analyzed by different types of chromatography techniques, such as gas chromatography and thin layer chromatography, and spectroscopy approaches, such as UV-Vis absorption, fluorescence, and infrared spectroscopies. Notably, these methods extensively use different types of chemometric tools for pattern recognition analysis to discriminate the biomarkers responses and generate a chemical fingerprint of the analyzed samples. The recent literature clearly confirms that these methods are highly sensitive, selective, and accurate in predicting the quality of different types of fruits, juices, and vegetables and their geographical origin. However, these methods are expensive, time-consuming, and lack real-time analysis owing to bulky instrumentation. In this context, we have highlighted the major research progress and commercialization made in the field of e-nose-based technologies, employing an array of gas sensors that are cheaper, portable, and capable of performing real-time analytical measurements in food quality assessment. Significant advancements have been reported in e-nose-based devices for food quality monitoring, which is also demonstrated by the commercialization of a sizable number of e-noses utilizing an MOS-based chemirestive gas sensors array. A review of the recent literature in this field reveals tremendous progress made in MOS-based chemiresistors for gas sensors and array-based e-nose, achieving high sensitivity, sub-ppm LOD, and stable responses. Coupled with different statistical analyses (PCA, LDA, and PLS-DA) and network analysis, these devices could classify the analyzed food aroma into identifiable biomarkers and thus predict the quality of the food. In addition to MOS-based gas sensors, significant advancements are reported in organic nanomaterials-based gas sensors in the e-nose. In particular, CPC-based sensing materials such as CNT or graphene-containing polymer nanocomposites and organic molecular materials such as porphyrins have drawn notable interests.

Despite substantial progress made in conventional analytical methods and vigorous scientific research ongoing in the emerging point-of-care technologies based on e-nose, there is still room for further improvements. Conventional methods based on optical spectroscopies have been underutilized in the food quality analysis field, especially methods based on UV-Vis and fluorescence spectroscopies, which are relatively much cheaper and more rapid than the chromatography-based methods. Currently, there are plenty of portable UV-Vis and fluorescence spectrophotometers available in the market (MRC Laboratories and Instruments, Shimadzu), which can be used in the future for a real-time analytical measurement of the food samples. Chromatography-based instrumentations have experienced significant miniaturization in recent years. For instance, portable GC-MS setups are now commercially available (Griffin G510 from Teledyne FLIR), which should be further improved to shorten the sampling and analysis times to make it suitable for real-time monitoring of food products. E-nose based technologies have multiple scopes for future improvements. In the gas sensors array module, efforts have to be made to improve the sensitivity, long-term stability, and selectivity of the sensors. This is because the concentration of VOC biomarkers to be detected is very low, usually in the sub-ppm range, necessitating the use of highly sensitive sensing materials and low-noise electronics. Moreover, an improvement in the selectivity would lower the complexity of the chemometrics data analysis and improve the resolution in the classification of the biomarkers. Additionally, efforts have to be made to develop low-cost sensing materials, as MOS-based gas sensors require complex synthesis procedures and high temperatures for operation. These challenges can be overcome by developing organic nanomaterials, which are relatively cheaper in cost, that can be solution processed on the sensor electrodes, and gas sensors based on these materials can be operated at room temperature. For instance, the diverse area of polymer nanocomposite-based materials, which have not been significantly explored in e-nose development applied for food quality assessment, can be an important area of interest. The selectivity of these materials towards a biomarker can be tuned by the right choice of polymers and a nanofiller. Thus, smart material engineering can allow for the design of highly selective gas sensors. Similarly, metal porphyrins and phthalocyanines-based materials have been extensively investigated for room temperature operating gas sensors for VOC, using different types of transducers (chemiresistive, gravimetric, optical, and electrochemical); however, they have not been applied to e-nose devices targeted for food quality evaluation. Thus, the application of different types of organic nanomaterials-based sensing materials in tandem with different types of transducers in the same e-nose device will potentially improve the selectivity of the device. Another pertinent area of development is the advancement of the existing chemometric tools to simplify the analysis, reduce the analysis time, and increase the accuracy. Moreover, the pattern recognition tools should be integrated with artificial intelligence to make the analytical procedure more accurate, rapid, and autonomous.

## Figures and Tables

**Table 1:** Volatile fingerprints of food products for quality monitoring.

Commodity	VOCs under Normal Storage	References
Pear	1-hexanol, bergamotol, butyl acetate, hexyl acetate, 2-decenal, (E,E)-α-Farnesene	[23,24]
Strawberry	Acetic acid, butyl ester, 2-butanoic acid, ethyl ester.	[25]
Pineapple	Citric acid, malic acid, iso-citric acid	[26,27]
Oranges, Mandarins	β-Terpineol, hexyl butanoate, α-Ionone, 2-Octenal, α-Guaiene	[16,28]
Banana	3-Methyl butyl butanoate, ethanol, Butyl butanoate, 3-Methyl butyl acetate	[29]
Papaya	Ethylene, 1-butanol, Methyl 2-hexenoate, 2-Heptanone, d-Limonene, α-Pinene	[30]
Garlic	3,4-dihydro-3-vinyl-1,2-dithiin and 2-vinyl-4H-1,3-dithiin	[31]
Peanut	Aldehydes, alcohols, ketones	[32]
Broccoli	Ethers, aldehydes, alkanes, ketones, esters, alcohols	[33]
Apple	Butyl acetate, butanol, methyl hexanoate, diethyl malate, acetic acid, hexanol	[14,34]
Potatoes	Decanal, 3,7–dimethyl–3–octanol, dodecanol, hexanal, 2-methyle propanal	[35]
Onion	Thiophene,2,4-dimethyl, dimethyl trisulfide, 3-thiophene carboxaldehyde, fully saturated thiosulfinates, mono-unsaturated thiosulfinates	[36]
Tomato	Hexanal, methanol, ethanol, acetaldehyde, pentanal, 1-nitro-pentane, 3-methylbutanal, 5-ethyl-2(5H)-furanone, hexanoic acid, 1-penten-3-one, 2-cyclohexene-1,4-dione	[37]
Carrot	monoterpenes, acetaldehyde, ethanol, β-myrcene, hexanal, octanal	[38]
Avocado	Methyle-acetate, acetaldehyde, hexanal, limonene, benzaldehyde, 1-pentene-3-one	[39]
Mango	3-Penten-2-ol, α-gurjunene, 2-pentanone, methyl 2-butenoate, terpinen-4-ol	[40]
Grapes	Ethyle acetate, hyxyl hexanoate, benzaldehyde, 1-heptanol, 1-butanol, 1-hexanol, acetic acid, geranic acid, D-limonene, linalool, nerol	[15]
Coffee bean	2,3-Butanedione, 3-methylpropanal, hexanal, 4-methyl-2-buteno-1-thiol, 4-methoxyphenol, 3-methylindole, mercapto-3-methylbutanol	[41]
Cocoa	Benzaldehyde, 2,3-Butanedione, Dimethyl disulfide, Methyl 2-phenylacetate, Dodecane, 2-n-Pentylfuran, 2,3,5,6-Tetramethylpyrazine	[42]
Guava	*Trans*-2-hexenol, α-Pinene, hexenyl acetate, limonene, Eucalyptol, Ethyl benzoate, α-Farnesene	[43]
Kiwi	Ethyl acetate, Methyl propionate, Ethyl benzoate, 1-Penten-3-ol, 1-pentanol, 1-octanol, α-Pinene, Camphene, Dimethyl sulfide, ethylbenzene, hexanoic acid	[44]

## Data Availability

No new data was created.
